# Current advances for omics-guided process optimization of microbial manufacturing

**DOI:** 10.1186/s40643-023-00647-2

**Published:** 2023-04-30

**Authors:** Shengtong Wan, Xin Liu, Wentao Sun, Bo Lv, Chun Li

**Affiliations:** 1 Key Laboratory of Medical Molecule Science and Pharmaceutical Engineering, Ministry of Industry and Information Technology, Institute of Biochemical Engineering, School of Chemistry and Chemical Engineering, Beijing Institute of Technology, Beijing, 100081 People’s Republic of China; 2 Department of Chemical Engineering, Tsinghua University, Beijing, China; 3 Key Lab for Industrial Biocatalysis, Ministry of Education, Tsinghua University, Beijing, China; 4 Center for Synthetic and Systems Biology, Tsinghua University, Beijing, China

**Keywords:** Microbial manufacturing, Omics, Process optimization, Fermentation performance

## Abstract

Currently, microbial manufacturing is widely used in various fields, such as food, medicine and energy, for its advantages of greenness and sustainable development. Process optimization is the committed step enabling the commercialization of microbial manufacturing products. However, the present optimization processes mainly rely on experience or trial-and-error method ignoring the intrinsic connection between cellular physiological requirement and production performance, so in many cases the productivity of microbial manufacturing could not been fully exploited at economically feasible cost. Recently, the rapid development of omics technologies facilitates the comprehensive analysis of microbial metabolism and fermentation performance from multi-levels of molecules, cells and microenvironment. The use of omics technologies makes the process optimization more explicit, boosting microbial manufacturing performance and bringing significant economic benefits and social value. In this paper, the traditional and omics technologies-guided process optimization of microbial manufacturing are systematically reviewed, and the future trend of process optimization is prospected.

## Introduction

Low-carbon and sustainable manufacturing has become the topical subject of global economic development and environmentally friendly microbial manufacturing has developed rapidly in the fields of food, medicine and energy, which bring huge economic effects and social value to the world (Bi et al. 2021; Hu et al. 2021; Shi et al. 2022).

Recent advances have been made in many commercial cases of microbial manufacturing using high-performance strains in artemisinin (Kung et al. 2018), farnesene (Liu et al. 2022), 1,3-propanediol (Zhu et al. 2021), succinic acid (Ahn et al. 2016) and other products. During the common process optimization, traditional methods and experimental design such as single factor experiment, orthogonal experiment, Plackett–Burman and Box–Behnken designs are extensively used to optimize the medium components and environmental factors directly. However, the intrinsic connection of cell metabolism and culture condition is not clear, and the optimization methods are often “black box” and time-consuming.

**Fig. 1:**
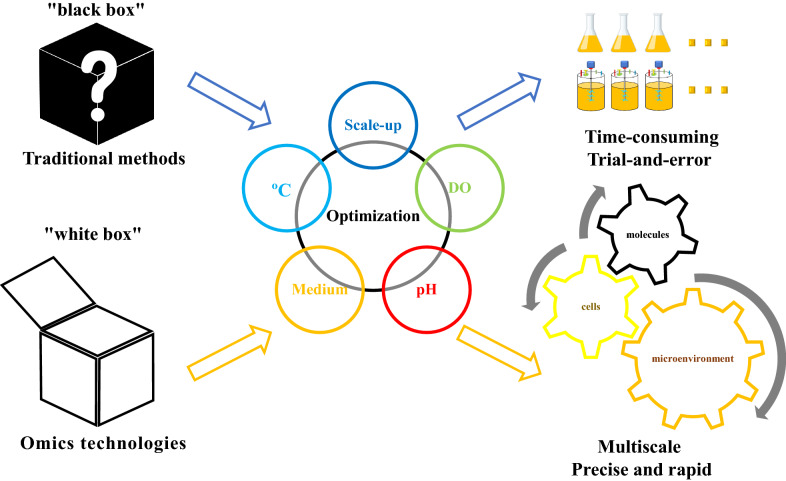
Comparison of microbial manufacturing process optimization using traditional methods and omics technologies

With the rapid development of omics technologies, the process optimization of microbial manufacturing also can be guided by omics, including genomics, transcriptomics, proteomics, metabolomics and the metabolic network model constructed on this basis. These technologies allow the overall analysis of transcription, translation and metabolism from the molecular level (Xu et al. 2018; Amer and Baidoo 2021).Through the omics analysis of different microbial phenotypes, we can truly gain insight into the changes of overall metabolism caused by the microenvironment changes, which help us explore the macro factors that affect the performance of microbial manufacturing from multiscale of molecular-cell–microenvironment and facilitate the realization of accurate and rapid optimization (Fig. 1).

In this paper, we have summarized the current strategies and successful cases of microbial manufacturing process optimization based on the long used experimental design and that guided by omics technologies, and then looked forward to the future trend of process optimization with greater productivity and lower cost.

## The development of microbial manufacturing

At present, many pharmaceutical intermediates, food additives and natural products are mainly produced by animal and plant extracts or chemical synthesis. The unsustainable production processes bring a lot of problems, such as waste of natural resources and serious environmental pollution (Sun et al. 2022; Ko et al. 2020). With the strengthening of human awareness of environmental protection, green and sustainable microbial manufacturing using microbial cell factories as production units and renewable resources as raw materials has been widely studied in the production of various kinds of natural and bulk chemicals (Zhu et al. 2020).

**Fig. 2:**
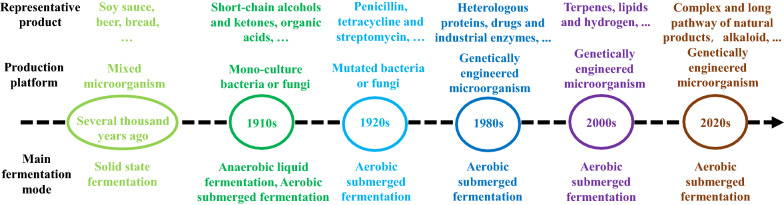
Main development history of microbial manufacturing

Actually, human beings have a long history of using microorganisms for daily life and production since 9000 years ago (Mcgovern et al. 2004). With the birth of modern microbiology and the maturity of fermentation technology, microbial manufacturing appeared at the beginning of the twentieth century. Due to research on glycolysis intermediates and the demand for explosives with the outbreak of World War I, the use of microorganisms to produce various organic acids, short-chain alcohols and ketones were developed. From 1920 to 1940, the discovery of penicillin and the great demand for antibiotics during the outbreak of World War II opened a new chapter in the history of microbial manufacturing with fast development in all aspects. Then, in 1980s, genetic engineering started to be used for modifying microorganisms at the genetic level to achieve the desired phenotypes, such as producing a variety of heterologous proteins, drugs and industrial enzymes. In the twenty-first century, the rapid development of synthetic biology facilitates the use of microbial cell factories to produce biofuels and chemicals and causes a new round of scientific and technological revolution in the world for green and sustainable development. Up to now, microbial cell factories have been used to produce various compounds including fuels, bulk chemicals, enzymes and natural products (Fig. 2) (Amer and Baidoo 2021; Buchholz and Collins 2013; Zhang et al. 2017, 2022a; Srinivasan and Smolke 2020).

Although microbial manufacturing can bring great social and economic benefits, but the output of many microbial products is still too low to meet the need of commercial production. Thus, the performance of microbial manufacturing needs to be improved and process optimization is a key factor, by which the optimization and scale-up of fermentation processes could improve the performance of high-performance strains with better productivity and lower cost (Son et al. 2023).

## Synthetic biology for high-performance strains

High-performance strain is the core of microbial manufacturing, and synthetic biology is an important tool for building high-performance strains. Although different cells, such as *Actinomycetes*, *Bacillus subtilis*, *Saccharomyces cerevisiae*, cyanobacteria and microalgae could be selected for microbial manufacturing, the synthetic biology strategies generally focus on the rational design of biosynthetic pathways for products, the construction and optimization of biosynthetic pathways, and the heterogenic expression of biosynthetic pathways. These strategies can be summarized as follows: new enzymes mining and synthesis pathways design, precursors enhancement and cofactors regeneration, protein engineering, weakening competitive pathways, balancing cell growth and production, and transport engineering to reduce the feedback inhibition and product cytotoxicity (Zhu et al. 2020; Bu et al. 2021; Carsanba et al. 2021; Choi et al. 2019, 2020; Zhang et al. 2022b; Aziz et al. 2020; Savakis and Hellingwerf 2015). The single or combined use of these strategies can effectively improve the performance of microbial manufacturing.

For example, Dai. et al. constructed the synthetic pathway of oleanolic acid in *S. cerevisiae* by introducing β-amyrin synthase, oleanolic acid synthase and so on from different plants; meanwhile, they overexpressed some enzymes in the pathway to increase the precursor supply, achieving heterologous biosynthesis of 21.4 mg/L oleanolic acid in *S. cerevisiae *(Dai et al. 2014). Zhang et al. combined site-directed mutagenesis to modify santalene synthases and increasing precursor supply in *Escherichia coli*, and the yield of α-santalene reached to 887.5 mg/L, which was about 140 times higher than that of the original strain (Zhang et al. 2022c). When using systems metabolic strategy in engineered *Pichia pastoris* for boosting S-adenosyl-l-methionine production, Qin et al. downregulated the cystathionine β-synthase gene using a weak promoter with overexpressed *GDH2*, leading to an increase of S-adenosyl-l-methionine titer from 1.13 to 2.71 g/L in flask (Qin et al. 2020). Besides, Steiger et al. reported the overexpression of heterologous enzymes CexA and an inducible expression system using *ptet-on*, resulting in 109 g/L citric acid production which is increased more than five folds than that of original strain (Steiger et al. 2019). These methods can effectively improve the productivity of microbial manufacturing, but to maximize the performance, the actual production process is often inseparable from the optimization of fermentation process and medium composition (Gong et al. 2016). Zhao et al. performed optimization of carbon source concentration in *Saccharomyces cerevisiae* medium. The results showed that oleanolic acid production was 606.9 ± 9.1 mg/L under 40 g/L glucose, which was 2.3 times higher than the original medium (Zhao et al. 2018). In another study, Wu et al. optimized the fermentation strategy of mycophenolic acid (MPA) from *Penicillium brevicompactum*. The results showed that MPA production increased by 58% compared with that of the original fermentation (Wu et al. 2022). These studies demonstrate the optimization of fermentation process and medium composition can effectively improve the production level.

## Application of traditional methods in the process optimization of microbial manufacturing

The traditional medium optimization always adopts changing one factor at a time (OFAT) to screen the best single factor at the initial stage in optimization. While the experimental design (DOE) based on statistical method allows the preliminary screening of multiple factors in the limited number of experiments and can overcome the limitations of OFAT method. For example, the response surface methodology (RMS), a mathematically model on the basis of the optimal single factors, considers the interaction between factors to predict the dependent variables. Because of the ease and convenience, these methodologies are still in use even today (Singh et al. 2017). The production capacity of microorganisms is closely related to the microenvironments in which the cells located. The microenvironments are influenced by the compositions of the medium (carbon source, nitrogen source, trace elements, etc.), parameters of fermentation (temperature, dissolved oxygen (DO), pH, etc.) and the fermentation strategies (batch fermentation, continuous fermentation and fed-batch fermentation) (Table 1). Controlling and regulating the microenvironment at different levels during fermentation process could greatly promote the production of target products at high titer, which is a prerequisite for the commercialization of microbial manufacturing (Rokem et al. 2007).

**Table 1:** Fermentation strategy of microbial manufacturing

Product group	Product	Strain	Carbon source	Nitrogen source	Temperature ( ℃)	DO	pH	Feedingstrategy	Fermentation mode	Biomass	Titer	References
Terpene compound	α-humulene	*Oleaginous yeast Candida*	Glucose	Peptone	30	> 20%	5.5	When the concentration of glucose fell below 5 g/L for the first time, high sugar solution was added	Fed-batch, two-phase fermentation	OD_600_: 470	4.115 g/L	Zhang et al. (2022d)
	α-santalene	*Escherichia coli*	Glucose, glycerol	Tryptone	37	-	7.0	Constant rate (80 mL/h) with glycerol mixture	Fed-batch, two-phase fermentation	OD_600_:250	2.916 g/L	Zhang et al. (2022c)
	β-amyrin	*Saccharomyces cerevisiae*	Glucose, ethanol	Peptone	30	> 40%	5.5	Three-stage feeding controlled by glucose and ethanol	Fed-batch	OD_600_:334	2.6 g/L	Du et al. (2022a)
	α-farnesene	*Saccharomyces cerevisiae*	Glucose	Peptone	30	–	5.5	When the initial carbon source was exhausted, glucose was fed to keep its concentration below 1.0 g/L	Fed-batch, two-phase fermentation	OD_600_:400	10.4 g/L	Wang et al. (2021)
	( +)-valencene	*Saccharomyces cerevisiae*	Glucose, sucrose	(NH_4_)_2_SO_4_	30	> 20%	5.0	-	Fed-batch, two-phase fermentation	DCW:105 g/L	16.6 g/L	Ye et al. (2022)
	(−)-eremophilene	*Saccharomyces cerevisiae*	Glucose, ethanol	(NH_4_)_2_SO_4_	30	10–30%	5.0	Two-stage feeding controlled by glucose and ethanol	Fed-batch, two-phase fermentation	OD_600_:425	34.6 g/L	Deng et al. (2022)
	Amorpha-4,11-diene	*Saccharomyces cerevisiae*	Glucose, ethanol	(NH_4_)_2_SO_4_	30	40%	5.0	Ethanol pulse feeding (10 g/L)	Fed-batch	OD_600_:195	4 g/L	Westfall et al. (2012)
	Artemisinin	*Saccharomyces cerevisiae*	Glucose, ethanol	(NH_4_)_2_SO_4_	30	40%	5.0	Ethanol pulse feeding (10 g/L)	Fed-batch, two-phase fermentation	–	25 g/L	Paddon et al. (2013)
	Geranylgeraniol	*Saccharomyces cerevisiae*	Glucose, ethanol	(NH_4_)_2_SO_4,_corn steep liquor	33	–	5.5	Glucose, glucose/ethanol mixed feed (5.8 g/h)	Fed-batch	–	3.31 g/L	Tokuhiro et al. (2009)
	Geraniol	*Saccharomyces cerevisiae*	Glucose, ethanol	Peptone	30	> 30%	5.7	Adding glucose or ethanol, glucose and ethanol concentrations were lower than 1 g/L and 5 g/L, respectively	Fed-batch, two-phase fermentation	OD_600_:85	1.68 g/L	Jiang et al. (2017)
	Geraniol	*Saccharomyces cerevisiae*	Glucose, ethanol	(NH_4_)_2_SO_4_	30	> 30%	5.0	Constant flow rate of 0.1 L/h plus 400 g/L ethanol	Fed-batch, two-phase fermentation	OD_600_:55	1.69 g/L	Zhao et al. 2017)
	Limonene	*Saccharomyces cerevisiae*	Glucose, ethanol	Peptone	30	-	-	Adding a certain amount of ethanol at 20 and 40 h	Fed-Batch Shake Flasks	OD_600_:33	0.918 g/L	Cheng et al. (2019)
Organic acids	Succinic acid	*Escherichia coli*	Glucose, galactose	Tryptone	37	-	7.0	-	Fed-batch, two-stage fermentation controlled by DO	DCW:2 g/L	22.4 g/L	Olajuyin et al. (2016)
	L-valine	*Escherichia coli*	Glucose	(NH_4_)_2_SO_4_	37	> 20%	7.0	Adding glucose solution at a constant rate to keep the concentration of residual sugar lower than 5 g/L	Fed-batch, two-stage aerobic and oxygen limitation fermentation	OD_600_:75	84 g/L	Hao et al. (2020)
	Citric acid	*Yarrowia lipolytica*	Glucose	(NH_4_)_2_SO_4_	30	50%	6.0	Glucose pulse feeding regulated by dissolved oxygen	Fed-batch, nutritionally limited fermentation	DCW:15 g/L	80–85 g/L	Kamzolova and Morgunov (2017)
	Pyruvic acid	*Candida glabrata*	Glucose	spent yeast cell dry powder	30	–	5.5	-	Batch	DCW:17.7 g/L	65.1 g/L	Lu et al. (2022)
Biobased chemicals	1,3‐Propanediol	*Klebsiella pneumoniae*	Glucose	NH_4_Cl	30	–	7.0	Intermittent addition of 500 g/L glucose solution with a concentration of 50 to 150 mM	Fed-batch	OD_600_:20	62 g/L	Lama et al. (2020)
	Polyhydroxybutyrates	*Bacillus megaterium*	Glucose	(NH_4_)_2_SO_4_	37	–	7.0	–	Batch	DCW:10.52 g/L	5.61 g/L	Mohanrasu et al. (2020)
	Hyaluronan	*Streptococcus zooepidemicus*	Glucose	Tryptone	37	–	7.0	–	Fed-batch, two-stage fermentation with pH and temperature control	–	4.75 g/L	Liu et al. (2018a)
	Fusaricidins	*Paenibacillus kribbensis* CU01	Glucose	NH_4_Cl	30	–	6.8	The process of continuous cultivation at a constant rate of 2.5 ml/min using a feed of fresh modified M9 medium	Continuous Fermentation	–	0.579 g/L	Ryu et al. (2020)
	Lipid	*Yarrowia lipolytica* SKY7	Glycerol	Tryptone	28	25–40%	6.5	When glycerol concentration reached below 5 g/L, a glycerol feed was added so that the glycerol concentration was 20–25 g/L	Fed-batch	DCW:51.67 g/L	19.47 g/L	Kumar et al. (2021)
	Recombinant antibody	*Escherichia coli*	Glucose	NH_4_Cl	30	30%	6.8	Constant flow rate of 0.1 g/L/h plus ethanol	Fed-batch, oxygen limitation fermentation	OD_600_:73	2.056 g/L	Biermann et al. (2013)

### Optimization of medium composition

Culture medium is the material basis for microbial growth and product synthesis (Singh et al. 2017), and its optimization is an essential process for large-scale production using microorganisms, which mainly includes the optimization in three aspects of carbon source, nitrogen source and trace elements. Carbon source is the major source of carbon skeleton and energy supply for microorganisms. Glucose is the most widely used carbon source in the fermentation process for its easy utilization and proper cost. In microbial manufacturing, glucose can be efficiently transformed into NAD(P)H cofactors and universal terpene precursors IPP and DMAPP, and it has been successfully used in the production of many natural terpene products including α-humulene (Zhang et al. 2022d), α-santalene (Scalcinati et al. 2012), ( +)-valencene (Ye et al. 2022), (−)-eremophilene (Deng et al. 2022). Due to the short metabolic pathway to converting ethanol in to acetyl-CoA, ethanol is usually added to the fermentation broth as a flow-through carbon source for boosting production (Dai et al. 2013). For example, using ethanol as the flow-through carbon source enhanced the yields of some products, such as amorpha-4,11-diene (Westfall et al. 2012), artemisinin (Paddon et al. 2013), β-amyrin (Du et al. 2022a) and geranylgeraniol (Tokuhiro et al. 2009).

Nitrogen source is the major material used to maintain cell growth and participate in the synthesis of nitrogen-containing compounds (Parente et al. 2018). In microbial manufacturing, microorganisms can utilize different kinds of nitrogen sources, such as amino acids (Brambilla et al. 2016), ammonium ions (Mendes-Ferreira et al. 2004), peptides (Kevvai et al. 2016), urea (Yang et al. 2021) and so on. Considering the cost and utilization, nitrogen sources commonly used in microbial fermentation mainly include (NH_4_)_2_SO_4_ (Mohanrasu et al. 2020), (NH_4_)_2_HPO_4_ (Sun et al. 2016), NH_4_Cl (Ryu et al. 2020), yeast extract (Goksungur and Guvenc 1997), peptone (Sun et al. 2021), corn steep liquor (Shen et al. 2016) and so on. Among them, nitrogen in inorganic nitrogen sources can be rapidly absorbed and utilized by microorganisms and leave acidic or alkaline substances in the fermentation broth, which usually changes the pH of fermentation broth (Lu et al. 2011). Thus, rational use of inorganic nitrogen source also plays a positive role in stabilizing and regulating pH during the fermentation process. The organic nitrogen sources including yeast extract, corn steep liquor, and peptone, not only provide nitrogen sources, but also provide inorganic salts, vitamins and growth factors (Lu et al. 2022), and the nitrogen utilization varies with different organic nitrogen sources during cell growth and product synthesis (Lu et al. 2011). Hence, utilization of multiple nitrogen sources is an efficient strategy to balance the growth and metabolite synthesis during fermentation for the purpose of improving the yield (Crepin et al. 2017).

The trace elements are indispensable nutrients and play an important role in microbial metabolism, moreover, the activators of many enzymes in cells (Locatelli et al. 2016; Palermo et al. 2021). Inorganic salts are the main form of trace elements (Rocha et al. 2019). Usually, the complex medium is rich in inorganic salts, but their content may not meet the requirements of rapid growth and metabolism of microorganisms, which will inevitably affect the fermentation performance (Coltin et al. 2022). For example, Kumar et al. studied purified glycerol as carbon and trace elements source for lipid and citric acid production using *Yarrowia lipolytica.* The results showed that the trace elements in purified glycerol were not enough for cell growth and lipid production, resulting in low biomass (27.67 g/L) and lipid titer (9.35 g/L). Thus, by external adding trace elements (in the form of phosphates and sulfates), a higher biomass (51.67 g/L) and lipid titer (19.47 g/L) were finally obtained (Kumar et al. 2021).

Meanwhile, trace elements also affect the formation of by-products during the fermentation process. Biermann et al. analyzed the trace element-associated response of recombinant antibody producing *Escherichia coli* to oxygen limitation. During fed-batch cultivation with provoked oxygen limitation, norleucine and norvaline accumulated as by-products only in the absence of molybdenum, nickel, and selenium. Opposite of this, the trace element supplemented fermentation showed significantly lower titer of these by-products. This could help to develop new strategy to avoid the formation of by-products (Biermann et al. 2013).

### Optimization of fermentation process

Fermentation conditions including temperature, dissolved oxygen, and pH are crucial parameters regulating the growth of microorganisms and the synthesis of products. However, the optimal fermentation parameters are different at various stages of fermentation and varies with different hosts. To overcome the limited performance of constant fermentation control, researchers have developed staged fermentation strategy to keep cells in a relatively favorable microenvironment at all stages of fermentation, allowing for optimal production performance (Burg et al. 2016).

For example, in the production of hyaluronic whose function depends on the molecular weight, Liu et al. developed a two-stage fermentation process to balance hyaluronic synthesis and its molecular weight increase in different stages of fermentation. In the first fermentation stage, the pH value was kept at 8.0 and the temperature was kept at 31 ℃ to promote the increase of molecular weight of hyaluronic, while in the second fermentation stage, the pH value and temperature were maintained at 7.0 and 37 ℃, respectively. Finally, the titer of hyaluronic increased from 3.58 g/L to 4.75 g/L (Liu et al. 2018a). In the production of L-valine in *Escherichia coli*, to enhance the supply of pyruvate precursors needed for L-valine synthesis, Hao et al. designed a two-stage fermentation controlled by DO to reduce the pyruvate consumption by TCA cycle. First, the aerobic fermentation led to rapid cell growth, and then limited DO level was used for rapid product accumulation. At last, L-valine production increased by twofold to 0.41 g/g glucose (Hao et al. 2020).

In addition, the optimization of the fermentation modes can also significantly improve microbial manufacturing performance, including the production of α-santalene (Zhang et al. 2022c), β-amyrin (Du et al. 2022a), geraniol (Jiang et al. 2017; Zhao et al. 2017) and limonene (Cheng et al. 2019). Currently, the commonly used fermentation modes are batch fermentation, continuous fermentation and fed-batch fermentation, in which fed-batch fermentation is used in most commercial production processes. The commonly used feeding strategies include **μ**-stat (Wang et al. 2017), DO-stat (Andrade et al. 2019), pH–stat (Kim et al. 2004), constant rate feeding (Zhu et al. 2015) and limitative feeding based on nutrient control (Ma et al. 2019) in fed-batch fermentation. Different feeding strategies have significant effects on microbial manufacturing performance. For example, Li et al. compared and analyzed the use of three feeding strategies of DO-stat, pH–stat and constant rate feeding in the production of β-alanine by *Saccharomyces cerevisiae*. In the constant rate feeding, the high consumption rate of glucose and rapid increase of biomass in the early stage of fermentation, resulting in insufficient carbon flux to the β-alanine synthesis. In the DO-stat feeding, the feedback of DO signal changes caused by cell metabolism was delayed to a certain extent, so that the cells would be in a certain starvation state. However, the pH–stat feeding strategy avoided excessive glucose consumption during fed-batch fermentation, resulting in the highest titer of β-alanine, which is 64.15 g/L (Li et al. 2022). Thus, the use of reasonable feeding strategy in fed-batch fermentation can greatly improve the titer and substrate conversion (Mears et al. 2017).

## Applications of omics technologies in the process optimization of microbial manufacturing

Although optimization of medium composition, fermentation condition, and fermentation mode by traditional methods can promote microbial manufacturing performance, this process is still empirical with many unknowns and requires a mass of trial-and-error experiments. Since the emergence of omics technology, it has been rapidly developed and become an important tool to reveal the molecular mechanism of life process (Dai and Shen 2022). Omics mainly includes genomics, transcriptomes, proteomics, metabolomics and the metabolic network model built on the basis of omics, which is a system biological analysis method based on high-throughput detection and bioinformatics technology. It can reflect the global transcription, translation and metabolism of organisms from the multi-scale and help determine the cell needs during the production process. At present, omics technologies are widely used in producing food, medicine and energy by microbial cell factories (Amer and Baidoo 2021).

**Fig. 3:**
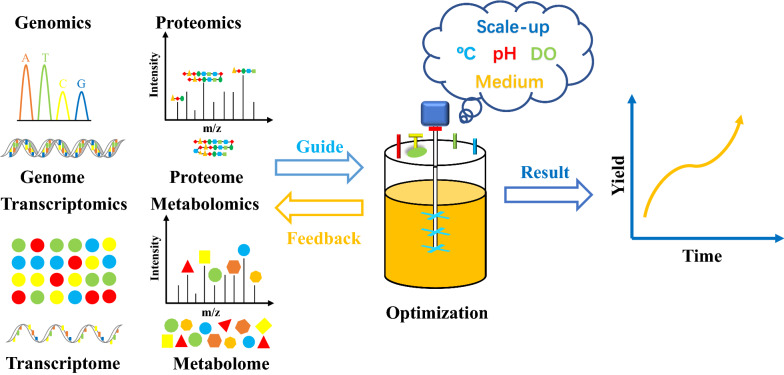
Optimization strategy of microbial manufacturing process guided by omics technologies

Genomic and transcriptomic analyses are essential in evaluating genome engineering contribution, allowing for the prediction of phenotypic traits and the optimal conditions for achieving high production performance. In addition, proteomics and metabolomics have gained significant attention for providing intracellular metabolic information that can reflect the function and phenotype of microorganisms (Amer and Baidoo 2021). These omics data can not only truly reflect the metabolism rearrangement caused by the microenvironment changes, but also help us explore the factors affecting the manufacturing performance of microorganisms at the microscopic level (temperature, dissolved oxygen, pH, precursor supply, substrate/product inhibition, etc.), achieving multi-scale process optimization simultaneously considering molecular-cellular-microenvironment and accelerating the realization of precise and rapid optimization. In recent years, omics technologies have been successfully applied to guide the optimization of microbial manufacturing process in optimization of medium composition, fermentation process and scale-up of fermentation process (Fig. 3) (Table 2) (Palazzotto and Weber 2018; Fondi and Lio 2015).

**Table 2:** Omics technologies-guided microbial manufacturing process optimization cases

Omics technology	Strain	Guiding role	Meaning	References
Metabolomics	*Corynebacterium glutamicum*	It found the bottleneck of carbon source utilization	Which laid a theoretical foundation for the subsequent use of pentose as a carbon source for fermentation	Kawaguchi et al. (2018)
	*Yarrowia Lipolytica*	To elucidate the effect of the C/N ratio medium on lipid production	It found that high C/N ratio medium promoted long-chain fatty acids synthesis better	Yun et al. (2018)
	*Chromochloris zofingiensis*	To elucidate the effect of the addition of gibberellic acid-3, sodium chloride and high C/N ratio on astaxanthin production	It provided the help for optimization of the medium composition for increase astaxanthin content	Chen et al. (2023)
	*Streptomyces*	To elucidate the molecular mechanism of pH on the synthesis of candicidin	The step-by-step control strategy of pH in fermentation processes was put forward	Liu et al. (2018b)
Transcriptomics	*Monascus*	It found that carbon starvation could cause stress response in cells, and direct acetyl-CoA pigments synthesis	To provide a direction for subsequent fermentation optimization to improve pigment yield	Yang et al. (2015)
	*Thraustochytrids*	To elucidate the regulatory mechanism of corn steep liquor for improving lipid and docosahexaenoic acid productivity	To provide a basis for using low-cost corn steep liquor as nitrogen source for fermentation	Wang et al. (2019)
	*P. purpureum*	To elucidate the metabolic mechanism of polysaccharides and PUFAs were significantly improved under nitrogen stress	which was helpful for the design of medium and process control during under nitrogen stress fermentation	Ji et al. (2021)
	*Saccharomyces cerevisiae*	To investigate the reasons for the significant difference on the carotenoid production in two different media (YPM, YPD)	It provided the key factors (Zn^2+^, Cu^2+^) in the optimization of medium for carotenoid production	Su et al. (2021)
	*Zymomonas mobilis*	The Mg^2+^ deficiency in the medium were majorly related to stress response and energy conservation	The concentration of Mg^2+^ in the nitrogen source was critical for cell growth and ethanol fermentation	Li et al. (2020)
	*Streptomyces*	pH shock increased yield by increasing ε-poly-L-lysine synthase activity and improving cellular respiration	It provided a reasonable explanation for the effect of pH on the synthesis of ε-poly-L-lysine	Pan et al. (2019)
Lipidomics	*Mortierella alpina*	To reveal the effects of types of nitrogen sources on the lipid metabolism	To provide an optimal direction for regulating the ratio of different components in lipid	Lu et al. (2019)
GSMN	*Nostoc sp.*	To elucidate PBPs biosynthesis's potential association in the cell	To provide a direction for subsequent fermentation optimization to improve PBPs yield	Saini et al. (2021)
	*P. thermoglucosidasius*	To resolve a previously unclear bottleneck in anaerobic fermentation	To identifying the minimal required supplemented nutrients needed to sustain anaerobic growth	Mol et al. (2021)
Comparative transcriptomics and lipidomics	*Aurantiochytrium* sp.	To elucidate the effects of different temperatures (5 °C, 15 °C) treatments on the biosynthesis of long-chain polyunsaturated fatty acids	The accumulation of DHA was increased under 5 °C, the yield of eicosadienoic acid (20:2) increased at 15 °C	Song et al. (2022)
Genomics, comparative transcriptomics and proteomics	*Rhodosporidium toruloides*	To elucidate the molecular mechanism of nitrogen-limited starvation on the accumulation of oil	which was helpful for the design of medium and process control during nitrogen-limited fermentation	Zhu et al. (2012)
Transcriptomics, proteomics, metabolomics	*Rhodosporidium toruloides*	To elucidate the molecular mechanism of phosphate-limited starvation on the accumulation of lipids	To provide new substances for nutritionally limited production	Wang et al. (2018)
Proteomics, metabolomics	*Phaffia rhodozyma*	It found that succinate increased carotenoid production mainly due to increased acetyl-CoA utilization and cellular respiration rate	As a reference for the production of carotenoids from non-fermentative carbon sources	Martinez-Moya et al. (2015)
Transcriptomics, metabolomics	*C. zofingiensis*	To elucidate the molecular mechanism of 0.2 M NaCl maximize both TAG and astaxanthin contents	It provided an accumulation of biodiesel in algae under salt stress theoretical basis	Mao et al. (2020)
	*Methanotrophs*	The changes of intracellular metabolism of cells to specific nutrient sources were determined	It provided a carbon source and nitrogen source optimization theoretical basis	Sugden et al. (2021)
	*R. glutinis* ZHK	To elucidate the effects of different temperatures (16 °C, 32 °C) treatments on the biosynthesis of carotenoids, lipids and EPSs	To provide theoretical basis for optimizing temperature of fermentation to control the type of products	Zhao and Li (2022)
	*Zymomonas mobilis*	To elucidate the effects of aerobic and anaerobic fermentation on type of products	which was beneficial to control oxygen content to improve accumulation of ethanol	Yang et al. (2009)
	*Rhodotorula* sp.	To elucidate the molecular mechanism that low levels of cytoplasmic glycerol limited lipid production	The addition of glycerol to the fed-batch increased cell mass and lipid production	Zhao et al. (2021)
Transcriptomics, proteomics	*Streptococcus thermophilus*	To elucidate most remarkable growth phase changes were related to heterofermentation, glycolysis and peptidoglycan biosynthesis	which was helpful for the design of medium and provided the theoretical basis for high density fermentation	Qiao et al. (2019)
	CHO cell	To elucidate the effects of different fermentation scales on cell productivity	The hypoxia was an important reason for limiting the scale-up of fermentation	Gao et al. (2016)

### Optimization of medium composition guided by omics technologies

Understanding the overall changes of cell metabolism during fermentation at the molecular level is critical for the rational design of culture medium (Qiao et al. 2019). The traditional medium optimization overlooks the changes of cellular metabolism during process optimization, and the optimization processes are often time-consuming (Batista and Fernandes 2015). Through omics analysis, we can understand the changes of microbial nutrients demand during cell growth and product synthesis so as to achieve the best state of fermentation production.

For example, the *Corynebacterium glutamicum* has priority in the utilization of glucose out in the carbon sources. Through the metabolomics analysis of cells using D-glucose and L-arabinose, Kawaguchi et al. found the bottleneck of carbon source utilization and greatly promoted the utilization rate of L-arabinose through modifying the intracellular metabolic pathway, which laid a theoretical foundation for the subsequent use of pentose as a carbon source in fermentation (Kawaguchi et al. 2018). The production of lipids by *Yarrowia lipolytica* is highly dependent on the carbon source in the culture medium. High C/N ratio medium with glycerol as carbon source can obtain higher lipid production than the low C/N ratio medium with glucose as carbon source. Through metabolomics analysis of *Yarrowia lipolytica* in media with different C/N ratios, Yun et al. found that glycerol promoted long-chain fatty acids (such as stearic acid, palmitic acid and arachidic acid) compared with glucose (Yun et al. 2018). Chen et al. performed metabolomic analysis for the heterotrophically grown *C. zofingiensis* strain with significantly increased astaxanthin production when added gibberellic acid-3, sodium chloride and high C/N ratio in the medium. The results showed that the enhancement of the glycolysis, pentose phosphate pathways and TCA cycle led to astaxanthin accumulation, which provided help for the subsequent optimization of the medium composition for increasing astaxanthin yield (Chen et al. 2023). In addition, when using *Monascus* to produce pigments, relatively low molecular weight slow-release carbon sources (starch, etc.) needed to be degraded into disaccharides or monosaccharides for the utilization by microorganisms. Although they will make cells in a carbon starvation state for a period of time, but the yield of pigments increased. Through transcriptomics analysis of *Monascus* with starch as carbon source, Yang et al. found that carbon starvation could cause stress response in cells, inhibiting central carbon metabolism and directing acetyl-CoA pigments synthesis. This strategy provided a direction for subsequent fermentation optimization to improve pigments yield (Yang et al. 2015).

In addition to carbon sources, omics analysis also provides insight into the intracellular metabolism affected by nitrogen sources which influence strain growth and product synthesis, and provides input for subsequent process optimization. Lu et al. conducted a lipidomic analysis of lipid production by the oleaginous fungus *Mortierella alpina* under four nitrogen sources: soybean meal, yeast extract, potassium nitrate and ammonium tartrate, and found that soybean meal and ammonium tartrate were beneficial to the accumulation of triglycerides, arachidonic acid and C_16-18_ fatty acids, respectively. Which provided an optimal direction for regulating the ratio of different components in lipid (Lu et al. 2019). Corn steep liquor is a by-product of industrial production of corn starch, and has been a frequently-used nitrogen source in microbial manufacturing due to its low cost, richness in organic nitrogen and multiple vitamins. Wang et al. found that a low-concentration corn steep liquor solution facilitated the docosahexaenoic acid production of *thraustochytrids*, and high-concentration of corn steep liquor led to increased biomass. The transcriptomic analysis was performed at high, normal, and low levels of corn steep liquor in culture medium to elucidate the low level of corn steep liquor affecting the perception and transduction of a limited-nitrogen signal and interactions between the transcription factors at the transcriptional level for improving lipid and docosahexaenoic acid productivity, providing new directions for subsequent optimization of production (Wang et al. 2019). The contents of total lipids and exopolysaccharides in *P. purpureum* were significantly improved under nitrogen stress. Ji et al. performed transcriptome analysis of *P. purpureum* under nitrogen deprivation. The results showed that the upregulated genes were closely related to the synthesis of GDP-Man and UDP-GlcNAc, suggesting that *P. purpureum* may regulate the composition and structure of polysaccharides to cope with nitrogen stress (Ji et al. 2021). Thus, the balance between cell growth and product synthesis can be regulated by controlling the types and concentrations of nitrogen source during the fermentation process to exert the microbial manufacturing performance more effectively.

Moreover, omics analysis facilitate the optimization of metal ions to improve microbial manufacturing performance (Ciosek et al. 2020). For example, Su et al. performed transcriptomic analysis for significant differences in the production of carotenoid by *Saccharomyces cerevisiae* in two different media YPM (modified by traditional methods) and YPD. The results showed that acetate transporter gene *ADY2* that was responsive for zinc ions was downregulated by 8 times, and the *CUP1-2* gene encoded copper metallothionein was upregulated about 94 times. After adding zinc ions, copper ions and simultaneous supplementation of zinc and copper ions in YPD medium, the yields of carotenoid were increased by 2.3 times, 9.2 times and 9.7 times, respectively. It provided the key factors for the subsequent optimization of the medium for carotenoid production (Su et al. 2021). Besides, Li et al. performed transcriptomic analysis for significant differences in the cell growth and production of ethanol by *Zymomonas mobilis* in two different media (RM, MM), and found that the lack of Mg^2+^ triggered stress responses and reduced the expression of genes involved in energy metabolism, which affected cell growth and product synthesis. Thus, the Mg^2+^ deficiency in the medium was majorly related to stress responses and energy conservation (Li et al. 2020). The importance of metal ions to cell growth and product synthesis suggests that they should be indispensable parameters for optimizing microbial medium (Kosaka et al. 2020).

Although genetic, transcriptional, protein and metabolic levels of cells can be analyzed separately using omics, single omics analysis is difficult to give a comprehensive and precise reflect of metabolism in cells. As a result, integrated multi-omics analysis was developed to comprehensively analyze molecular mechanism at different levels of cells. For example, using genomic, comparative transcriptomic and comparative proteomic analyses, Zhu et al. found that nitrogen-limited starvation is an important reason for oil accumulation in the oleaginous fungi *Rhodosporidium toruloides* due to nitrogen-limited condition correlated with the induction of lipogenesis and nitrogenous compound recycling, which provided a theoretical basis for the design of medium and process control during nitrogen-limited oil-producing fermentation (Zhu et al. 2012). Besides, lipid production can also be facilitated by limiting other nutrients, such as sulfates, phosphates, inorganic carbon and iron (Granger et al. 1993; Wu et al. 2010, 2011; Hu et al. 2020). For example, Wang et al. analyzed the transcriptional, protein and metabolic levels of cells under both phosphate-limited and sufficient conditions and found that phosphate-limited conditions promoted phosphate metabolism, TAG biosynthesis and the downregulated TCA cycle, resulting in an elevated flux of carbon to lipid synthesis and facilitating lipid production (Wang et al. 2018). Mao et al. performed comparative transcriptomes and metabolomes analyses and demonstrated 0.2 M NaCl concentration was optimal for maximizing both TAG and astaxanthin production in *C. zofingiensis* under salt stress. The study found that the increased accumulation of TAG due to the carbon shunt from starch and enhanced acetyl-CoA production by the coordinated up-regulation in multiple pathways, and the diverted carotenoid flux from lutein to astaxanthin due to the up-regulation of lycopene beta cyclase and beta-carotenoid ketolase, providing the theoretical basis for the accumulation of biodiesel in algae under salt stress (Mao et al. 2020). In addition, the multi-omics analysis explains the effect of carbon source on microbial manufacturing performance very well. Carbon source can affect the carotenoid production of *Phaffia rhodozyma*, which could achieve higher production under non-fermentable carbon source. Based on the proteomic and metabolomic analyses of cells under fermentable carbon source (glucose) and non-fermentable carbon source (succinate), Martinez-Moya et al. found that succinate increased carotenoid production mainly due to increasing acetyl-CoA utilization and cellular respiration rate, and producing more reactive oxygen species (Martinez-Moya et al. 2015). Sugden et al. performed transcriptome and metabolome analyses of *Methanotrophs* using methane or methanol as carbon source and nitrate or ammonium as nitrogen source. The study displayed that ammonium upregulated hydroxylamine dehydrogenase and overall central metabolic activity, while nitrate upregulated genes for nitrate assimilation and conversion. These results suggested the changes of intracellular metabolism caused by specific nutrient sources, and provided valuable theoretical basis for future studies on process optimization (Sugden et al. 2021).

In recent years, genome-scale metabolic models (GSMs) have gained in popularity in the study of the effects of genetic and environmental perturbations on cell metabolism and cell growth (Kim et al. 2015). With the ability to provide a mechanistic link between metabolic phenotypes and cellular genotypes, GSMs have been employed as a tool for predicting possible metabolic limitations and optimizing fermentation conditions and medium composition. Metabolic flux analysis (MFA) is a widely used approach to estimating intracellular fluxes under a defined metabolic network (Singh and Lercher 2020; Veras et al. 2019). Saini et al. developed a genome-scale metabolic network (GSMN) for *Nostoc* sp. to investigate the potential association of phycobiliproteins (PBPs) biosynthesis with cellular processes. The MFA suggested that metabolic processes associated with aminolevtulinic acid precursor of PBPs and energy supply processes were critical for increasing PBPs production (Saini et al. 2021). In another study, Mol et al. established a GSMN of *P. thermoglucosidasius* to optimize strain productivity. Hypothesis-driven experiments based on the GSMN revealed a previously unclear bottleneck in anaerobic fermentation, and identified the minimal supplemented nutrients (thiamin, biotin, and iron (III)) necessary to sustain anaerobic growth (Mol et al. 2021). These studies demonstrate the importance of GSMs and MFA in guiding microbial manufacturing performance.

### Optimization of fermentation process guided by omics technologies

The traditional experience-based process optimization is a "black box", which overlooks the change of intracellular metabolism with process parameters (Mandenius and Brundin 2008; Yazici et al. 2021). Omics technologies are recently used for turning the "black box" to "white box", and greatly improve the efficiency of fermentation processes optimization.

Temperature is one of the important parameters affecting the productivity of microbial manufacturing. It affects cell morphology, cell membrane composition, enzyme expression, and activity, which are essential for cell growth and the synthesis of products during fermentation (Knapp and Huang 2022; Li et al. 2021). Since temperature usually increases during the fermentation process, heat tolerance become one of the important robustness traits of industrial strains, the quality of heat resistance has important value for saving industrial production costs (Li et al. 2021; Xu et al. 2020; Hu et al. 2022). Song et al. carried out comparative transcriptomic and lipidomic analyses of the biosynthesis of long-chain polyunsaturated fatty acids (PUFAs) in *Aurantiochytrium* sp. under different temperatures (5 °C, 15 °C). The study found that the accumulation of docosahexaenoic acid (DHA) increased by 1.25-fold due to upregulated expression of genes involved in fatty acid synthase (FAS) and polyketide synthase (PKS) pathways under 5 °C, and the yield of eicosadienoic acid (20:2) increased by twofold due to upregulated FAS and fatty elongase 3 involved in the FAS pathway at 15 °C (Song et al. 2022). Besides, Zhao et al. studied the effects of low (16 °C) and high (32 °C) temperature treatments on the biosynthesis of carotenoids, lipids, and exopolysaccharides (EPSs) in oleaginous red yeast *R. glutinis* ZHK. Integrated transcriptomics and metabolomics analysis revealed that low-temperature treatment significantly increased EPSs production, while high-temperature treatment significantly increased lipids and carotenoids production by affecting the activity of key enzymes in the TCA cycle and the expression of carotenoids, lipids and EPSs biosynthetic genes in *R. glutinis* ZHK (Zhao and Li 2022). Therefore, the yield and type of the final product can be controlled by stage-temperature control in microbial manufacturing.pH significantly affects the cell growth and product synthesis, and the optimal pH value usually varies during the fermentation process. Liu et al. carried out metabolome analysis of the biosynthesis of candicidin in *Streptomyces* under fixed pHs (6.8, 7.8) and pH stepwise control (6.8–7.8). The study found that the yield of candicidin under pH stepwise control was almost twofold than that under fixed pHs due to the abundant precursors, malonyl-CoA and methylmalonyl-CoA were guaranteed to the rapid and continuous candicidin biosynthesis during the late stage of fermentation (Liu et al. 2018b). In the early research of producing **ε**-poly-L-lysine by *Streptomyces albulus*, pH shock could significantly increase the yield of ε-poly-L-lysine, but the mechanism of this process was unclear. The transcriptome analysis of *Streptomyces albulus* under pH shock showed that the increase of **ε**-poly-L-lysine synthase activity and the improvement of cell respiration were the main factors for the increased yield, which provided a reasonable explanation for the effect of pH on the synthesis of ε-poly-L-lysine (Pan et al. 2019). In addition to pH, the demand for dissolved oxygen of microorganisms in the fermentation process is also changing all the time. Through the transcriptome and metabolome analysis of *Zymomonas mobilis* ZM4 under aerobic and anaerobic conditions, the better fermentation performance under anaerobic condition was verified due to the 30-fold increased transcription level of key genes related to ethanol synthesis than that under aerobic condition. Moreover, larger amounts of byproducts were detected under aerobic conditions. Therefore, it was beneficial to control oxygen content during the fermentation process to reduce the production of byproducts and improve the accumulation of ethanol (Yang et al. 2009).

High density fermentation is always used to increase the specific production rate, reduce the reactor volume and the cost in commercial production (Shiloach and Fass 2005; Scheel et al. 2021). By combining transcriptome and proteome analysis, Qiao et al. studied the changes in the transcriptional and protein levels of *Streptococcus thermophilus* during its fermentation process. The study found that different amino acids (AAs) conversion occurred during the late-lag, late-exponential and stationary phases of fermentation, indicating that the intracellular concentrations of these AAs could be insufficient in these phases. Meanwhile, the cells uptake did not meet their requirements due to the imbalance nutrient components in the medium, limiting the increase of biomass during the fermentation processes. This study provided a theoretical basis for high density fermentation of *Streptococcus thermophilus* and reasonably design of the culture medium and fermentation process parameters for industrial application (Qiao et al. 2019). Besides, through transcriptome and metabolome analysis of the original strain *Rhodotorula* sp*.* and its mutants, Zhao et al. found the increased glycerol transport and utilization during the period of rapid bacterial growth, and the downregulated mannitol biosynthesis during the lipid production phase led to better lipid productivity, suggesting that low level of cytoplasmic glycerol may limit lipid production and supplementation of glycerol during fermentation production could increase both bacterial mass and lipid production(Zhao et al. 2021).

### Scale-up of fermentation process guided by omics technologies

The scale-up of fermentation process is the only way to realize the commercialization of microbial manufacturing. Due to the lack of understanding and experience for the scale-up of fermentation process, this process has the characteristics, such as high risk, high investment and long cycle, which hinders the further development of microbial manufacturing (Crater and Lievense 2018; Du et al. 2022b). Most omics analyses for pre-process optimization are performed at the level of shaking flasks culture (Tai et al. 2005). Due to the uncontrollable pH, insufficient mixing, low dissolved oxygen and evaporation of water in shake flasks, the data of the shake flasks usually do not match the fermenter, resulting in many obstacles and unknown factors in the scale-up of fermentation process (Singh et al. 2017). Meanwhile, the different scales of fermentation may bring about differences in the spatiotemporal distribution of aeration and feeding, resulting in heterogeneity of substrates, dissolved oxygen, products and harmful substances in the reactor, which leads to drastic changes in cell metabolism. Thus, the fermentation production performance usually decreases with the enlarged scale (Wang et al. 2020; Gao et al. 2016).

Since the metabolic situation in cells under different fermentation scales will change drastically due to the changes in the microenvironment. Thus, the comparative omics analysis of different fermentation scales could help understand the relationship between the microenvironment and internal metabolism of cells in the fermenter during the scale-up of fermentation process and explore the metabolic bottlenecks that affecting cell growth and product synthesis due to microenvironment changes, so as to achieve a reasonable scale-up of fermentation (Zou et al. 2012). In addition, Wang et al. used multi-omics integrative analysis and computer-aided design to combine cell reaction dynamics and computational fluid dynamics to provide a good prediction and evaluation method for the scale-up of fermentation (Wang et al. 2020). Tang et al. designed a metabolic model by coupling computational fluid dynamics with cell reaction dynamics to simulate the fermentation performance of *Penicillium chrysogenum* at different fermentation scales, and provided a rational strategy for the scale-up fermentation (Tang et al. 2017).

The oxygen uptake rate is an important parameter in the scale-up of fermentation process. Gao et al. studied the significant difference of Chinese hamster ovary (CHO) cells productivity between a 20-L bench-top scale bioreactor and a 5-KL production scale under seemingly identical process parameters. The integrated metabolomics and proteomics data revealed that the excess ROS produced in the 5-kL compared to the 20-L scale due to intermittent hypoxia in the industrial scale, which may lead to CHO cells apoptosis and affect productivity (Gao et al. 2016). Thus, it is necessary to ensure a sufficient supply of oxygen when scale-up of fermentation to improve the microbial manufacturing performance. Zou et al. simulated the oxygen uptake and oxygen transfer effects on the erythromycin production under the fermentation scales of 50 L and 132 m^3^ by computational fluid dynamics and found that a relatively high oxygen uptake rate (OUR) in the early phase of fermentation favored the biosynthesis of erythromycin, and the decrease of oxygen transfer rate (OTR) in 132 m^3^ fermenter was the main reason affecting the physiological metabolism of cells and biosynthesis of erythromycin (Zou et al. 2012). By optimizing oxygen uptake and oxygen transfer rate, the fermentation process could be scaled up effectively.

## Conclusions and prospects

As the world pays more and more attention to environmental protection and greenness development, microbial manufacturing plays an increasingly important role in human life and production for the advantages of environmental protection and sustainable production. However, the lack of ideal fermentation process remains a major obstacle to reducing cost and boosting the capacity of microbial manufacturing for the eventual commercial production.

The traditional experience-based process optimization often is "black box" and time-consuming. With the rapid development of omics technologies, the use of multi-omics technologies to analyze the fermentation processes from different levels, including transcription, protein and metabolism, is helpful to understand the response relationship between the internal metabolism of microorganisms and the extracellular microenvironment during the fermentation processes, which provides clear direction for microbial manufacturing optimization. However, the metabolic bottleneck gained by diving into different levels of omics data in process optimization is still at its nascent stage, and the omics analysis will bring massive amounts of data, including the uptake and utilization of nutrients, cell growth, synthesis of product, efflux of product and so on. Hence, how to quickly and accurately capture the metabolic bottleneck from the sea of data by machine learning would be an important direction for future research in the process optimization of microbial manufacturing.

In addition, in terms of optimizing microbial manufacturing process to improve the yield and reduce cost, the selection and design of fermentation reactors, pretreatment of substrates, wastewater treatment, and downstream separation and purification should also be considered, so that the performance of microbial manufacturing can be fully improved and benefit human beings.

## Data Availability

Not applicable.
